# The *Burkholderia cenocepacia* OmpA-like protein BCAL2958: identification, characterization, and detection of anti-BCAL2958 antibodies in serum from *B. cepacia* complex-infected Cystic Fibrosis patients

**DOI:** 10.1186/s13568-016-0212-1

**Published:** 2016-06-21

**Authors:** Sílvia A. Sousa, Mostafa Morad, Joana R. Feliciano, Tiago Pita, Soad Nady, Rehab E. El-Hennamy, Mona Abdel-Rahman, José Cavaco, Luísa Pereira, Celeste Barreto, Jorge H. Leitão

**Affiliations:** iBB-Institute for Bioengineering and Biosciences, Department of Bioengineering, Instituto Superior Técnico, Universidade de Lisboa, Av. Rovisco Pais, Torre Sul, Piso 6, 1049-001 Lisbon, Portugal; Zoology Department, Faculty of Science, Helwan University, Cairo, Egypt; Cystic Fibrosis Center, Department of Paediatrics, Hospital de D. Estefânia, Centro Hospitalar de Lisboa Central, Rua Jacinta Marto, 1169-045 Lisbon, Portugal; Cystic Fibrosis Center, Department of Paediatrics, Hospital de Santa Maria, Centro Hospitalar Lisboa Norte, Av. Prof. Egas Moniz, 1649-028 Lisbon, Portugal

**Keywords:** *Burkholderia cepacia* complex (Bcc), Cystic fibrosis (CF), OmpA-like proteins, Bcc positive CF serum samples, Neutrophil activation

## Abstract

Respiratory infections by bacteria of the *Burkholderia cepacia* complex (Bcc) remain an important cause of morbidity and mortality among cystic fibrosis patients, highlighting the need for novel therapeutic strategies. In the present work we have studied the *B. cenocepacia* protein BCAL2958, a member of the OmpA-like family of proteins, demonstrated as highly immunogenic in other pathogens and capable of eliciting strong host immune responses. The encoding gene was cloned and the protein, produced as a 6× His-tagged derivative, was used to produce polyclonal antibodies. Bioinformatics analyses led to the identification of sequences encoding proteins with a similarity higher than 96 % to BCAL2958 in all the publicly available Bcc genomes. Furthermore, using the antibody it was experimentally demonstrated that this protein is produced by all the 12 analyzed strains from 7 Bcc species. In addition, results are also presented showing the presence of anti-BCAL2958 antibodies in sera from cystic fibrosis patients with a clinical record of respiratory infection by Bcc, and the ability of the purified protein to in vitro stimulate neutrophils. The widespread production of the protein by Bcc members, together with its ability to stimulate the immune system and the detection of circulating antibodies in patients with a documented record of Bcc infection strongly suggest that the protein is a potential candidate for usage in preventive therapies of infections by Bcc.

## Introduction

The thickened mucus layer of the cystic fibrosis (CF) lung, with enhanced airway surface liquid volume absorption, reduced clearance and hypoxia gradients provides a habitat for opportunistic pathogens (Worlitzsch et al. 2002). This environment promotes host neutrophil invasion with subsequent release of oxidants and proteases, such as myeloperoxidase (MPO) and neutrophil elastase (NE), causing progressive and continued lung tissue damage (Downey et al. 2009; Cohen-Cymberknoh et al. 2013; Watt et al. 2005; Worlitzsch et al. 1998). Secreted and cell surface bacterial proteins are essential in mediating infection and inflammation processes by the host. Binding of these proteins to host cells is now recognized as triggering inflammatory responses leading to the release of host cytokines like TNF-α and various interleukins, to combat the pathogen (Watt et al. 2005).

The *Burkholderia cepacia* complex (Bcc) comprises important opportunistic pathogens capable of causing life threatening lung infections among cystic fibrosis (CF) patients (Leitão et al. 2010; Drevinek and Mahenthiralingam 2010). Despite advances in therapy, chronic infections with Bcc remains a problematic issue because these pathogens are very difficult to eradicate and have been associated with a faster decline of lung function, increased morbidity and mortality of patients (Mahenthiralingam et al. 2005; Hauser et al. 2011). No effective therapies are currently available to eradicate Bcc bacteria from CF patients, as these are inherently resistant to the majority of antimicrobials clinically available (Leitão et al. 2008; Regan and Bhatt 2014). Therefore, therapeutic strategies that protect patients against early Bcc lung infections represent attractive measures to control these infections.

In a previous work from our research group, the screening of a *B. cenocepacia* J2315-derived plasposon mutant library allowed the identification of *BCAL2958* as a putative virulence determinant in the infection model *Caenorhabditis elegans* (Sousa et al. 2008 and unpublished results). The gene was identified after the rescue of a DNA fragment containing the inserted plasposon and surrounding DNA sequences, using previously described methods (Sousa et al. 2008; Ramos et al. 2010). Analysis of the nucleotide sequence revealed that the plasposon was inserted in the intergenic region upstream BCAL2958 which encodes an outer membrane protein A (OmpA)-like protein. OmpA-like proteins from other gram-negative bacterial species are surface exposed proteins that have been shown to occur at high copy number. These proteins have been associated with virulence, being involved in adhesion and invasion of host cells, induction of cell death, serum and antimicrobial resistance, and immune evasion (Krishnan and Prasadarao 2012; Smani et al. 2014). OmpA-like proteins from several pathogens, like the OprF from the CF pathogen *Pseudomonas aeruginosa,* have been associated with respiratory epithelial adhesion with cell activation through intracellular signalling pathways that results in release of cytokines and chemokines (Fito-Boncompte et al. 2011).

Since OmpA-like proteins have been pointed out as good candidates for vaccine development (Krishnan and Prasadarao 2012), we decided to investigate the immunogenic properties of *B. cenocepacia* J2315 BCAL2958 protein, envisaging its future exploitation as a immunoprotectant against Bcc infections. In the present work we report bioinformatics studies predicting the occurrence of immunogenic epitopes on the protein, and on the conservation and occurrence of genes encoding OmpA-like proteins in members of the Bcc with completed and publicly available genome sequences. These bioinformatics studies were complemented by experimental data demonstrating that the protein is expressed by several strains based on western-blot carried out using an antibody raised against *B. cenocepacia* J2315 BCAL2958. Results are also presented showing the presence of antibodies against BCAL2958 in blood sera from 4 CF patients with a known record of Bcc infection. Results on the ability of the protein to stimulate human neutrophils, as evidenced by increased release of TNFα, elastase, myeloperoxidase, hydrogen peroxide, nitric oxide and catalase by neutrophils upon exposure to BCAL 2958 are also presented. Results here presented strongly suggest that *B. cenocepacia* J2315 *BCAL2958*, encoding an OmpA-like protein, is a strong immunostimulant produced by Bcc, with potential usage as an immunoprotectant against Bcc infections.

## Materials and methods

### Bacterial strains, plasmids, and culture conditions

The bacterial strains and plasmids used in this work are listed in Table 1. When in use, *Bcc* strains were maintained in PIA (Pseudomonas Isolation Agar, BD) plates. *Escherichia coli* strains were maintained in LB (Lennox broth, Sigma) agar plates, supplemented with 150 µg ampicillin ml^−1^, when appropriate. Unless otherwise stated, liquid cultures were carried out at 37 °C in LB liquid medium supplemented with the appropriate antibiotics, with orbital agitation (250 rev min^−1^). Bacterial growth was followed by measuring the cultures optical density at 640 nm (OD_640_).

**Table 1:** Bacterial strains and plasmids used in this work

Strain or plasmid	Genotype or description	References or source
Strains		
*B. cepacia* LMG18821	Cystic fibrosis clinical isolate (Australia), MLST ST5	Mahenthiralingam et al. (2000)
*B. contaminans* IST408	Cystic fibrosis clinical isolate (Portugal), MLST ST96	Richau et al. (2000)
*B. multivorans* LMG16660	Govan C1576, Cystic fibrosis clinical isolate (UK), Glasgow epidemic reference, MLST ST27	Mahenthiralingam et al. (2000)
*B. multivorans* LMG18825	Ryley CF-A1-1, Cystic fibrosis clinical isolate (UK), South Wales outbreak, MLST ST15	Mahenthiralingam et al. (2000)
*B. cenocepacia* LMG16656	Govan J2315, Cystic fibrosis clinical isolate (UK), ET12 lineage reference strain, genome sequenced, MLST ST28	Govan and Deretic (1996)
*B. cenocepacia* R-1448	Cystic fibrosis clinical isolate (Canada)	Mil-Homens et al. (2010)
*B. cenocepacia* LMG18829	Speert PC184, Cystic fibrosis clinical isolate (USA), Midwest strain, MLST ST40	Mahenthiralingam et al. (2000)
*B. cenocepacia* R-4194	Cystic fibrosis clinical isolate	Mil-Homens et al. (2010)
*B. cenocepacia* LMG16654	Govan J415; Cystic fibrosis clinical isolate (UK), MLST ST34	Mahenthiralingam et al. (2000)
*B. stabilis* LMG14294	Cystic fibrosis clinical isolate (Belgium), MLST ST50	Mahenthiralingam et al. (2000)
*B. vietnamiensis* R-5143	Cystic fibrosis clinical isolate	Prof. Gerd Döring
*B. dolosa* LMG18944	LiPuma PC688, Cystic fibrosis clinical isolate (USA)	Coenye et al. (2001)
*Escherichia coli* DH5α	F^−^ endA1 glnV44 thi-1 recA1 relA1 gyrA96 deoR nupG Φ80d*lacZ*ΔM15 Δ(*lacZYA*-*argF*)U169, hsdR17(r_K_^−^ m_K_^+^), λ–	Invitrogen
*E. coli* BL21 (DE3)	F^−^ *ompT* *hsd*S_B_ (r_B_^−^m_B_^−^) dcm gal λ(DE3).	Stratagene
Plasmids		
pET23a+	Cloning/expression vector, T7 promoter, C-terminal 6× His-Tag, Ap^r^	Novagen
pSAS6	pET23a^+^ with *BCAL2958* gene cloned downstream of T7 promoter	This study

### Molecular biology techniques

Total DNA was extracted from cells harvested from exponentially-growing liquid cultures of *B. cenocepacia* strain J2315 using the High Pure PCR Template Preparation Kit (Roche). Plasmid isolation and purification (Zymo Research), DNA amplification (Finnzymes), restriction and T4 DNA ligation (Fermentas), agarose gel electrophoresis, SDS-PAGE and *E. coli* transformation were carried out using standard procedures (Sambrook and Russel 2001). The primers used for amplification of *BCAL2958* were UP-BCAL2958 (5′-TTGGATCCATGAATAAA CTTT-3′) and LW-BCAL2958 (5′-AAAAGCTTGTTTGCCGGAAC-3′), containing the *Bam*HI and *Hind*III restriction sites (underlined), respectively, at their 5′end. Primers were designed based on the genome sequence of *B. cenocepacia* J2315 (available at the Sanger Institute Homepage; http://www.sanger.ac.uk/Projects/ B_cenocepacia), using the software Oligo Primer Analysis v. 4, as previously described (Sousa et al. 2014).

### Cloning and overexpression of *B. cenocepacia* J2315 BCAL2958

Plasmid pET23a^+^ and the 682 bp PCR product obtained using primers UP- BCAL2958 and LW- BCAL2958 were digested with the restriction enzymes *Bam*HI and *Hin*dIII. The *BCAL2958* fragment was ligated into the *Bam*HI/*Hin*dIII digested pET23a^+^, yielding pSAS6. pSAS6 construction was confirmed by sequencing. This plasmid allows the controlled expression of *BCAL2958* by the T7 promoter upon isopropyl β-D-thiogalactoside (IPTG) induction, producing a BCAL2958 derivative with a 6× His-tag at the C-terminus. Plasmid pSAS6 was transformed into *E. coli* BL21 (DE3) and the 6× His-tagged protein was overexpressed by growing transformed *E. coli* BL21 (DE3) in 250 ml of LB liquid medium (supplemented with 150 µg/ml ampicillin) at 30 °C and with orbital agitation (250 rpm). When the culture reached an OD_640_ of 0.6, 0.4 mM IPTG (final concentration) was added and the culture was further incubated for 2 h at 30 °C, 250 rpm. 6× His-tagged BCAL2985 overproduction was assessed by SDS-PAGE analysis and immunoblot experiments using a monoclonal anti-polyhistidine peroxidase conjugate clone HIS-1 antibody (diluted 1:2000, SIGMA) as previously described (Sousa et al. 2013). Bacteria were then harvested by centrifugation for 5 min at 7000×*g* and 4 °C and the resulting pellet was resuspended in 8 ml sonication buffer (20 mM sodium phosphate, 500 mM NaCl, 20 mM Imidazole, pH 7.4). This cell suspension was aliquoted and stored at −80 °C until further processing.

### Purification of *B. cenocepacia* J2315 6× His-tagged BCAL2958

Bacterial cell suspensions were lysed by ultrasonic vibration with a Branson sonifier 250 (Branson), using 5 sonication cycles of 30 s each at 50 % duty cycle. When processing cell suspensions to obtain the 6× His-tagged BCAL2958, 2 % (V/V) Triton X-100 and 0.5 mM phenylmethylsulfonyl fluoride (PMSF) were added prior to the last two sonication cycles. After sonication, non-soluble cell debris were removed by centrifugation at 12,000×*g* for 30 min at 4 °C. The cleared supernatants were collected to new tubes and kept at 4 °C.

The 6× His-tagged protein BCAL2958 was purified by affinity chromatography using a HisTrap FF column (GE Healthcare). After the initial equilibration of the column with 10 ml of Start buffer [sodium phosphate buffer 1×, pH 7.4 (20 mM sodium phosphate, 500 mM NaCl); 20 mM Imidazole; 0.1 % (V/V) Triton X-100; 0.5 mM PMSF], the 6× His-tagged BCAL2958 protein was eluted with Start buffer containing increasing imidazole concentrations (50, 70, 90, 150, 250, and 500 mM). Aliquots (1 ml) of the collected fractions of each protein were analysed by SDS-PAGE, and those containing the purified proteins were dialysed overnight at 4 °C in a 10 kDa cutoff Slide-A-Lyzer Dialysis Cassette (Pierce) against sodium phosphate buffer 1× (pH 7.4). Protein concentration was estimated by the method of Bradford (Bradford 1976), with bovine serum albumin fraction V (BSA, Nzytech) as standard.

In order to produce polyclonal antibodies against 6× His-tagged BCAL2958, endotoxin contaminations were removed from the protein purified samples using the Detoxi-Gel ™ endotoxin removing gel (Thermo Scientific) following the supplier´s instructions and eluting protein samples with 1× Phosphate Buffered Saline (PBS) (137 mM NaCl, 2.7 mM KCl, 10 mM Na_2_PO_4_, 2.4 mM KH_2_PO_4_). The level of endotoxin in the purified 6× His-tagged BCAL2958 protein was estimated using the Pierce LAL chromogenic endotoxin quantitation kit (Thermo Scientific) according to the manufacturer instructions. Purified samples with endotoxin levels below 0.2 EU/ml were used. Production and purification of a polyclonal goat antibody anti-6× His-tagged BCAL2958 were performed by the commercial company SICGEN (Portugal) after receiving the protein purified as described above.

### Human serum samples

The serum samples S1 and S2 were collected from 2 CF patients infected with Bcc bacteria who attend the Hospital Santa Maria (Lisbon, Portugal), while serum samples S3 and S4 were obtained from two CF patients infected with Bcc who attended the Hospital de D. Estefânia (Lisbon, Portugal). Upon blood processing and serum recovery, serum samples were stored at −80 °C until further use. A pool of human blood serum from healthy persons, used as control, was obtained commercially (Sigma).

### CF patients blood sera immunoreactivity against the BCAL2958 protein

Purified 6× His-tagged BCAL2958 and BSA (used as a negative control, Nzytech) were loaded into 12.5 % SDS-PAGE gels and electrophoresed for 1 h at 150 V using standard procedures (Sambrook and Russel 2001). The gels were then incubated in transfer buffer (48 mM Tris, 39 mM glycin, 20 % (V/V) methanol, 0.04 % (W/V) SDS, pH 9.2) for 15 min and the proteins electrotransferred to nitrocellulose (NC) membranes (PALL corporation) using a Trans-Blot® SD (BIORAD) device apparatus at 15 mA for 1 h. After protein transfer, NC membranes were blocked overnight at 4 °C with 5 % (W/V) skim milk (DIFCO) in PBS 1×. Membranes were then probed with serum samples from CF patients (1:2000 dilution) or with a pool of human sera from healthy donors (1: 2000 dilution, SIGMA), for 3 h at room temperature. Membranes were washed with PBS 1× containing Tween 0.05 % (V/V), and subsequently incubated with a secondary antibody horseradish peroxidase (HRP)-conjugated Rabbit anti-Human IgG (1:5000 dilution, SANTA CRUZ biotechnology) for 1 h at room temperature. After removal of the secondary antibody and wash with PBS 1×/Tween 0.05 % (V/V), membranes were treated with the peroxidase substrate ECL (Sigma) and signals were detected using the FUSION Solo apparatus (Vilber Lourmat).

### Enzyme-linked immunosorbent assay (ELISA)

IgG levels against purified 6× His-tagged BCAL2958 in sera from CF patients with clinical history of Bcc was determined by enzyme-linked immunosorbent assay (ELISA). The OmpA was prepared at 2 µg/ml in 100 mM sodium carbonate buffer (pH 9.6) and 100 µl was applied per well to 96-wells ELISA plates (Greiner Microlon 600, Greiner Bio-One) and incubated overnight at 4 °C. The plates were blocked with 250 µl of 3 % BSA/PBS 1× overnight at 4 °C. Serum samples were serially diluted (1:100 to 1:100,000) in PBS 1 × supplemented with 3 % BSA and 0.05 % Tween. The diluted serum was added to the plates and they were incubated 2 h at 25 °C. Then, the plates were washed with PBS 1× containing 0.05 % Tween and were incubated with 100 µl of HRP-conjugated rabbit anti-Human IgG (SANTA CRUZ Biotechnology) antibody at 1:3000 in PBS supplemented with 3 % BSA and 0.05 % Tween. The plates were incubated 1 h at 25 °C. After washing the plates with PBS 1× containing 0.05 % Tween, it was added 100 µl of the peroxidase substrate 3,3′,5,5′-tetramethylbenzidine (TMB, SIGMA). After 20 min at 25 °C, the reaction was stopped by addition of 100 µl of 0.5 M H_2_SO_4_. The plates were read at 450 nm in the SPECTROstar Nano microplate reader (BMG LABTECH). A pool of sera from healthy humans (Sigma) was used as control. Internal positive- and negative-controls were included in each plate. All serum samples were analyzed in triplicate in two independent experiments, and the mean values were calculated.

Serum antibody concentrations were defined as endpoint titers and were calculated as the reciprocal of the highest serum dilution producing an OD_450_ above the cutoff value. The cutoff value was determined as the mean OD450 nm of the corresponding dilution of control sera plus 3 standard deviations. A titer of ≥1000 was considered positive for the ELISA.

### Western blot analysis of BCAL2958 expression by *Burkholderia cepacia* complex bacteria

A volume of the total cell extracts corresponding to 1 ml aliquot of a culture with an OD_640_ of 0.6 was dissolved in 40 µl of sample buffer [100 mM Tris base pH 6.8, 4 % (W/V) SDS, 20 % (V/V) glycerol, 0.2 % (W/V) bromophenol blue, 200 mM DTT], incubated for 5 min at 100 °C, and separated by 12.5 % SDS-PAGE. After electrophoresis, proteins were electrotransferred onto NC membranes (PALL corporation) using a Trans-Blot® SD (BIORAD), as described above. Then, the membranes were blocked with 5 % (W/V) skimmed milk (DIFCO) in PBS 1×, overnight at 4 °C. The membrane was then probed with the primary Goat antibody anti-BCAL2958 (1:3000 dilution, SICGEN) for 2 h at room temperature. Probing with the secondary antibody HRP—conjugated Mouse anti-Goat IgG (1:10,000 dilution, SANTA CRUZ biotechnology) was carried out for 1 h at room temperature. The membranes were treated with the peroxidase substrate ECL (Sigma). The chemiluminescence signals were detected using the FUSION Solo device (Vilber Lourmat).

### Isolation and purification of human neutrophils from blood

Neutrophils were obtained from human blood samples, collected from 8 adult healthy male volunteers (mean age 29 ± 4 years).

Neutrophils were isolated from EDTA anti-coagulated venous blood as described previously (Costa et al. 2006) with some modifications. Briefly, the blood was centrifuged using Ficol Hypaque (Amersham Pharmacia, Piscataway, NJ, USA) density gradient at 1500 rpm (HERMLE, USA) for 25 min to remove mononuclear cells. Then, red blood cells were lysed using ACK lysis buffer (Sigma, St. Louis, MO, USA). The neutrophils suspension was centrifuged at 2000 rpm (HERMLE, USA) for 10 min, the supernatant was discarded and cells were washed with Dulbecco’s modified eagle medium (DMEM) media (Lonza, Belgium). Finally, neutrophils were resuspended in DMEM and 10 % FBS (HyClone, UK) and cells were counted. Resulting neutrophil preparations were >98 % pure, as assessed by flow cytometry. More than 95 % of the neutrophils were viable as measured by trypan blue dye (ADWIC, Egypt) exclusion.

### Activation of neutrophils by OmpA

Isolated neutrophils (1 × 10^5^ cells/well) were activated by incubation with OmpA (2 µg/ml, as optimized). 100 ng/ml Lipopolysaccharide (Sigma-Aldrich, Germany) was used as a positive control. Supernatant was collected after 1, 2, 4, 8, and 12 h, for measuring MPO, TNF-α, Elastase, Hydrogen peroxide and Catalase, using ELISA methodologies and the Griess reagent to measure NO.

### Neutrophil mediators assessment

Nitric oxide (NO) was assessed using the Griess reagent based on methods previously described (Green et al. 1982). Hydrogen peroxide (H_2_O_2_) was measured using a colorimetric kit (Bio-diagnostic, Egypt) as previously described (Fossati et al. 1980). Catalase was measured using a colorimetric kit (Bio-diagnostic, Egypt) according to Aebi (1984**).** Human myeloperoxidase (MPO), Tumor necrosis factor-α (TNF-α) and Neutrophil elastase were measured using the ELISA kits MPO (Boster Immunoleader, USA), Human TNF-α (Boster Immunoleader, USA) and elastase (ASSAY PRO, USA), respectively, according to the manufacturer’s instructions. Non-activated neutrophils were used as negative controls.

### Bioinformatics analyses

Nucleotide and predicted amino acid sequences were analysed using bioinformatics tools resident at the National Center for Biotechnology Information (NCBI) and the ExPASy-Prosite websites. Searches for homologous sequences within the genomes of *B. cenocepacia* J2315 and other *Burkholderia* strains were carried out using the databases Integrated Microbial Genomes and *Burkholderia* Genome Database (Markowitz et al. 2014; Winsor et al. 2008). The prediction of B cell epitopes was performed using the BepiPred Linear Epitope Prediction method, resident at immune epitope database (IEDB) analysis resource (Larsen et al. 2006). A window size of 7 and a threshold of 0.35 were used. The threshold 0.35 was used because it is the point at which sensitivity/specificity is maximized in BepiPred (Larsen et al. 2006). Amino acid sequence alignments were generated using the CLUSTAL Omega Alignment (Sievers et al. 2011).

### Statistical analysis

Statistical analysis was performed using GraphPad Prism software. Paired Student’s t test between different treatments as well as ANOVA among different activation time points were analysed. The data obtained were represented as mean ± S.D. Results with a P value <0.05 were considered significant.

## Results

### Identification of *B. cenocepacia* J2315 *BCAL2958*, a member of the OmpA family

The *B. cenocepacia* J2315 gene *BCAL2958* was initially identified as a putative virulence determinant during the screening of a plasposon-derived mutant library performed with *Caenorhabditis elegans* as an infection model (Sousa et al. 2008). During the screening, a mutant strain named SJ2, impaired in its ability to kill the nematodes, was retained for further characterization. Based on methodologies previously described (Sousa et al. 2008), a plasmid harbouring the chromosomal DNA sequences flanking the inserted plasposon was retrieved from *B. cenocepacia* SJ2 mutant.

**Fig. 1:**
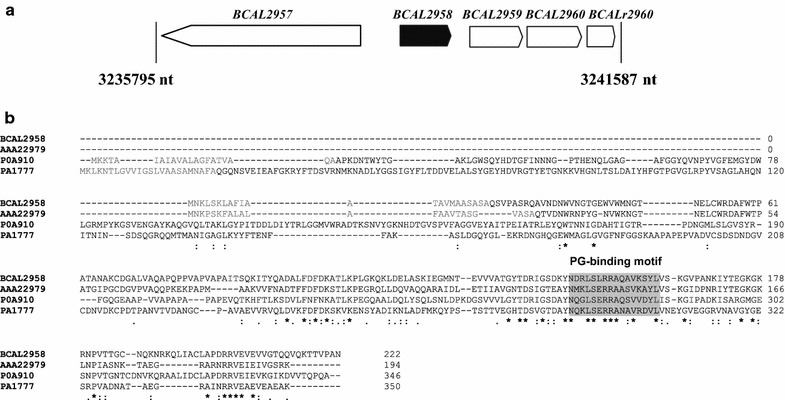
BCAL2958 is a putative OmpA-like protein. **a** Genetic organization of the *BCAL2958* gene locus. Open reading frames BCAL2957 (GyrA, DNA gyrase subunit A), BCAL2958 (OmpA, outer membrane protein A family protein), BCAL2959 (UbiG, 3-demethylubiquinone-9 3-demethyltransferase), BCAL2960 (Gph, putative 2-phosphoglycolate phosphatase), and BCALr2960 (SsrA, transfer-messenger RNA) are represented in scale; **b** Alignment of amino acid sequences of the OmpA-like proteins BCAL2958 from *B. cenocepacia* J2315, AAA22979 from *Bordetella avium*, P0A910 from *E. coli* K12 and PA1777 from *P. aeruginosa* PA01. The amino acid residues of the signal sequence are in *light grey*. The peptidoglycan (PG)-binding motif is *boxed* in *grey*. *Asterisks* indicate the amino acid residues that are identical in all the proteins; one or two *dots* indicate semi-conserved or conserved substitutions, respectively

**Fig. 2:**
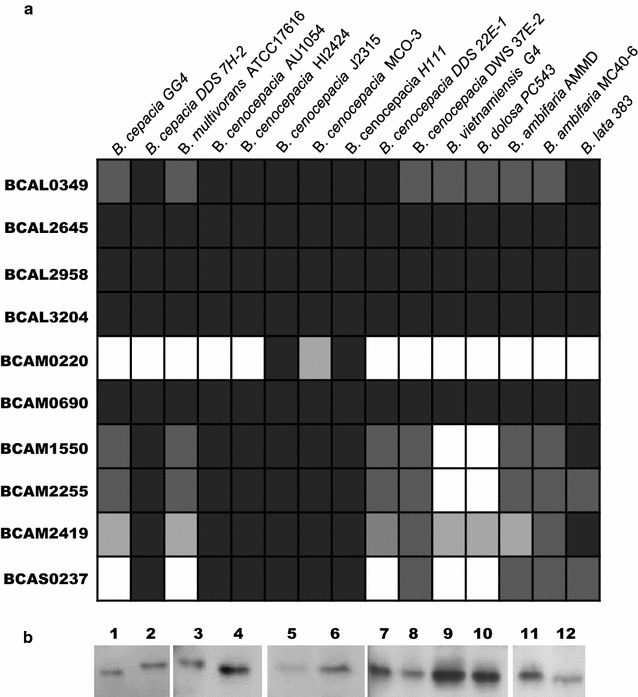
BCAL2958 is commonly expressed by Bcc strains. **a** Bioinformatics analysis of the presence, in the complete genome of 15 Bcc strains, of orthologues of the 10 proteins with the PF00691 domain identified in the *B. cenocepacia* J2315 genome. The % of identity of the orthologues is indicated as follows: higher than 90 % (*filled square*), higher than 80 % (*filled square*), higher than 70 % (*filled square*), or absence of the gene (*open square*); **b** Western blotting of Bcc strains probed with the Goat anti-BCAL2958 antibody. *Lanes* *1*—*B. cenocepacia* J2315; *2*—*B. contaminans* IST408; *3*—*B. vietnamiensis* R-5143; *4*—*B. cenocepacia* R-1448; *5*—*B. cepacia* LMG18821; *6*—*B. dolosa* LMG18944; *7*—*B. cenocepacia* R-4194; *8*—*B. multivorans* LMG16660; *9*—*B. multivorans* LMG18825; *10*—*B. cenocepacia* LMG18829; *11*—*B. cenocepacia* LMG16654; *12*—*B. stabilis* LMG14294

Analysis of the nucleotide sequence and searches for homologous sequences within the genome of *B. cenocepacia* J2315 revealed that the plasposon was inserted in the intergenic region upstream *BCAL2958* encoding an OmpA (outer membrane protein A)-like protein (Fig. 1a). The deduced amino acid sequence of BCAL2958 was found to contain the PFAM00691 motif (Fig. 1b), thus being a putative member of the OmpA family of proteins. The fact that several OmpA-like proteins from other bacterial pathogens have been regarded as potential vaccines prompted us to investigate *B. cenocepacia* J2315 BCAL2958 as a future vaccine or vaccine component (Pore and Chakrabarti 2013; Jeannin et al. 2002; Krishnan and Prasadarao 2012). First, we have performed bioinformatics analyses in order to gain further evidence of the presence of homologs within genomes of other Bcc bacteria, and also to other members of the *Burkholderia* genus. These analyses revealed a total of 10 putative OmpA-like proteins, unevenly distributed within the analysed genomes (Fig. 2a; Table 2) that included the following publicly available genomes: *B. cepacia* GG4 and DDS 7H-2; *B. multivorans* ATCC17616; *B. cenocepacia* strains AU1054; HI2424, J2315, MCO-3, H111, DDS 22E-1 and DWS 37E-2; *B. vietnamiensis* G4; *B. dolosa* PC543, *B. ambifaria* strains AMMD and MCO-40-6; and *B. lata* 383. The amino acid sequences of BCAL2958 homologs were ≥91 % identical and were present in all the genomes analysed, as well as those of BCAL2645, BCAL3204 and BCAM0690 (Fig. 2a, Table 2). BCAL0349 and BCAM2419 homologs were also present in all the genomes analysed, with amino acid identity percentages of at least 84 and 74 %, respectively (Fig. 2a; Table 2). The other four OmpA family members identified in our survey (BCAM0220, BCAM1550, BCAM2255 and BCAS0237) were absent, at different extent, in some Bcc genomes (Fig. 2a; Table 2).

**Table 2:** Analysis of the conservation in Bcc and predicted immunogenicity of *B. cenocepacia* J2315 OmpA-like proteins (PF00691)

Protein	Domains^a^	MW^b^	Signal sequence^c^	Conservation^d^	B cell epitopes average (predicted peptides)^e^
BCAL0349	BON (PF04972)	32.8	1–36 (SPI)	>84 % in Bcc	0.498 (13)
	OmpA (PF00691)			>72 % in *Burkholderia* genera	
				<66 % with other bacteria	
BCAL2645	Gly-zipper YMGG (PF13441)	21.6	1–21 (SPII)	>94 % in Bcc	0.433 (11)
	OmpA (PF00691)			>71 % in *Burkholderia* genera	
				<60 % with other bacteria	
BCAL2958	OmpA (PF00691)	23.9	1–22 (SPI)	>96 % in Bcc	0.362 (11)
				>87 % in *Burkholderia* genera	
				<77 % with other bacteria	
BCAL3204	OmpA (PF00691)	18.7	1–20 (SPII)	>91 % in Bcc	0.336 (9)
				>82 % in *Burkholderia* genera	
				<70 % with other bacteria	
BCAM0220	OmpA (PF00691)	25.3	1–22 (SPII)	Not conserved	0.369 (12)
BCAM0690	OmpA (PF00691)	22.6	NP	>92 % in Bcc	0.083 (11)
				>74 % in *Burkholderia* genera	
				<84 % with other bacteria	
BCAM1550	OmpA (PF00691)	17.7	1–18 (SPII)	Not conserved	0.319 (10)
BCAM2255	OmpA (PF00691)	18.3	1–26 (SPII)	Not conserved	0.120 (5)
BCAM2419	OmpA (PF00691)	22.9	1–17 (SPII)	>74 % in Bcc	0.363 (12)
				>35 % in *Burkholderia* genera	
				<36 % with other bacteria	
BCAS0237	OmpA (PF00691)	23.1	1–17 (SPII)	Not conserved	0.382 (13)

^a^ http://pfam.xfam.org/

^b^ http://web.expasy.org/protparam/

^c^ http://www.cbs.dtu.dk/services/LipoP/. *SPI* signal peptide (signal peptidase I); *SPII* lipoprotein signal peptide (signal peptidase II); *NP* no signal peptide

^d^ http://blast.ncbi.nlm.nih.gov/Blast.cgi

^e^ http://tools.immuneepitope.org/bcell/. Bepipred Linear Epitope Prediction method

In addition to the in silico results showing that BCAL2958 is encoded within all the Bcc genomes examined, we have also tested the expression of the protein by a panel of 12 Bcc strains by western-blot techniques. The panel included the following Bcc isolates from CF patients with different geographic origins: *B. contaminans* IST408; *B. cenocepacia* strains J2315, R-1448, R-4194, LMG18829, and LMG16654; *B. multivorans* LMG16660, and LMG18825; *B. cepacia* LMG18821; *B. stabilis* LMG14294; *B. vietnamiensis* R-5143; *B. dolosa* LMG18944 (kindly provided by Prof. Peter Vandamme, U. Ghent, Belgium). A goat polyclonal antibody commercially raised against *B. cenocepacia* J2315 BCAL2958 was used. A fraction containing total proteins from the *B. cenocepacia* J2315 strain was used as positive control in the assay. This analysis revealed that the antibody was able to detect the protein OmpA in all Bcc strains tested, revealing that this protein is commonly expressed in Bcc strains (Fig. 2b).

Analysis of the BCAL2958 amino acid sequence with ProtParam revealed that this protein has a predicted molecular weight of 23.9 kDa and a theoretical pI of 9.45. A signal peptide was predicted by LipoP 1.0 Server, with the cleavage site at the amino acid residue Ala^22^ (Table 2; Fig. 1b). The use of the CLUSTAL Omega bioinformatics tool revealed an amino acid identity of BCAL2958 of 52.06, 39.62, and 25 %, respectively, with the already described OmpA-like proteins from *Bordetella avium*, *E. coli* K12, and *P. aeruginosa* PAO1 (Fig. 1b) (Gentry-Weeks et al. 1992; Arora et al. 2001; Sugawara et al. 2006). The inspection of the amino acid sequence also revealed the presence of the peptidoglycan (PG) binding motif NX_2_LSX_2_RAX_2_VX_3_L in the C-terminal domain of BCAL2958, spanning residues 146–161 (Fig. 1b). This motif is typical of OmpA-like proteins, and is usually located in the periplasm (Smith et al. 2007). The N-terminal region of BCAL2958 was predicted as variable, with a particularly low identity in the case of the OmpA-like proteins from *E. coli* K12 and *P. aeruginosa* PAO1 (Fig. 1b).

### Cloning, expression and purification of BCAL2958

**Fig. 3:**
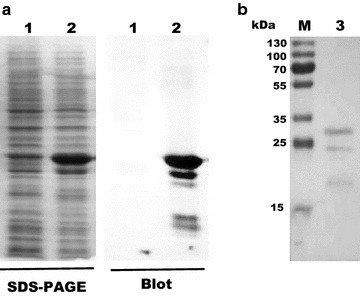
Expression and purification of BCAL2958 and analysis of the specificity of a goat antibody against BCAL2958. **a** Analysis of the specificity of a goat antibody against BCAL2958 by Western-blot. *Lanes* *1*—Total proteins from non-induced *E. coli* BL21 (DE3); *2*—total proteins from IPTG induced *E. coli* BL21 (DE3). **b** Purification analysis of the recombinant protein BCAL2958 from *E. coli* BL21 (DE3) by SDS-PAGE. *Lanes* *M*—PageRuler Plus Prestained Protein Ladder (Thermo Scientific); *3*—purified recombinant BCAL2958 protein

The 682 bp PCR fragment corresponding to the *BCAL2958* gene of *B. cenocepacia* J2315 was amplified using primers UP-BCAL2958 and LW-BCAL2958 (Table 1) and cloned into the expression vector pET23a + under the control of the T7 promoter, creating pSAS6 (Table 1). For the overexpression of the protein as a 6× His-tagged derivative, plasmid pSAS6 was transformed into *E. coli* BL21 (DE3) and the overexpression was induced by addition of 0.4 mM IPTG. The overproduced 6× His-tagged BCAL2958 protein was analysed by SDS-PAGE, followed by Western blot using an antibody specific for the BCAL2958 protein. This analysis revealed 3 different molecular weight forms of the protein, with estimated molecular masses of approximately 29.5, 25.5, and 19.2 kDa (Fig. 3), in agreement with previously reported observations for other OmpA-like proteins (Subramaniam et al. 2000) The first two values are in agreement with the predicted molecular masses of 25.5 and 23.3 kDa for the native protein with a 6× His-tag, of the pre-protein containing the signal peptide, and the mature protein without the signal peptide, respectively. No protein was detected by Western blot when using the cell extract prepared from *E. coli* cells grown without IPTG induction (Fig. 3a).

The recombinant protein was then purified to homogeneity by nickel affinity chromatography and the fractions containing the purified his-tagged protein were dialysed overnight against appropriate storage buffers and further studied. After purification, three forms of the protein were still apparent in the gel (Fig. 3b). The multiple bands could be due to different stages of processing of the protein, as previously described for other OmpA proteins (Gentry-Weeks et al. 1992; Subramaniam et al. 2000): (1) pro-OmpA, OmpA precursor that contains the signal peptide and is located in the cytoplasm or associated with the cytoplasmic membrane; (2) imp-OmpA, immature processed OmpA without the signal peptide and located in the periplasm or attached to the inner face of the outer membrane, and (3) mature OmpA. The molecular mass determined by SDS-PAGE of the OmpA protein is larger than the predicted molecular mass of the deduced amino acid sequence of OmpA. The small discrepancy between the apparent molecular mass determined by SDS-PAGE and that predicted for the protein has been reported in other outer membrane proteins (Manchur et al. 2011).

### The BCAL2958 protein is immunoreactive with sera from CF patients infected with Bcc

**Fig. 4:**
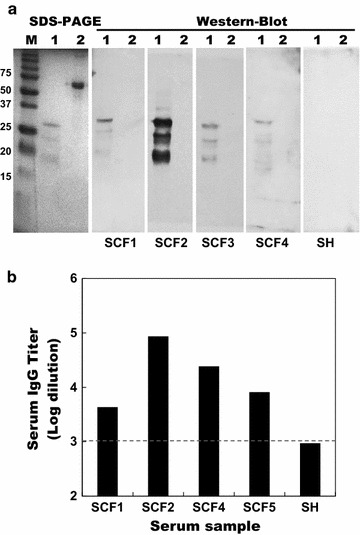
The OmpA-like BCAL2958 protein is immunoreactive with sera from CF patients infected with Bcc. **a** Western blotting of the purified recombinant protein BCAL2958 from *B. cenocepacia* J2315 probed with the Human serum samples SCF1, SCF2, SCF3 and SCF4 from CF patients infected with Bcc bacteria, or a pool of human serum sample from healthy donors SH. The BSA protein was used in the assay as a negative control. *Lanes* *M*—Precision Plus Protein™ Dual Xtra Standard (BIO-RAD); *1*—purified recombinant BCAL2958 protein; *2*—albumin bovine fraction V (BSA, Nzytech). **b** IgG antibody levels present on sera from CF patients infected with Bcc (SCF1, 2, 3 and 4) and in healthy individuals (SH) against BCAL2958 protein. Serum antibody concentrations were defined as endpoint titers and were calculated as the reciprocal of the highest serum dilution producing an OD450 nm above cutoff value. The cutoff value was determined as the mean OD450 nm of the corresponding dilution of control sera plus 3 standard deviations (*dashed line*). A titer of ≥1000 was considered as positive

To examine whether the BCAL2958 protein is capable of inducing an immune response in CF patients during infection with Bcc, we performed the detection of IgG antibodies against the protein in 4 serum samples collected from CF patients with culture-confirmed Bcc infections. The purified 6× His-tagged BCAL2958 protein reacted with all the serum samples (Fig. 4a), suggesting that this protein is exposed to the immunological system of the CF patients during the infection and is immunogenic. Bovine serum albumin fraction V was used as negative control (Fig. 4a). No reactivity of BCAL2958 protein was observed when using a sample of a pool of serum from healthy individuals (Fig. 4). The IgG antibody titers of each serum sample was determined by ELISA and revealed that all the samples from CF patients infected with Bcc had IgG titers higher than 4300, while the sample of a pool of serum from healthy individuals presented IgG titers below the threshold (Fig. 4b).

### The OmpA-like protein BCAL2958 interferes with neutrophils activity

Neutrophils are the first line of innate immune defense against infectious diseases (Kumar and Sharma 2010). Additionally, activated neutrophils regulate immune response by providing signals for the activation and maturation of both macrophages and dendritic cells (DCs) (Chertov et al. 1997, Bennouna et al. 2003). So, neutrophils can act as a transport vehicle for intracellular pathogens and deliver antigens to DCs, and thus play an important role in activation of T cell immune response by DCs (Megiovanni et al. 2006). Therefore, in this study we analyzed the response of neutrophils to the BCAL2958 protein by measuring the tumor necrosis factor alpha (TNFα), neutrophil elastase (NE), myeloperoxidase (MPO), hydrogen peroxide (H_2_O_2_), nitric oxide (NO) and catalase levels.

**Fig. 5:**
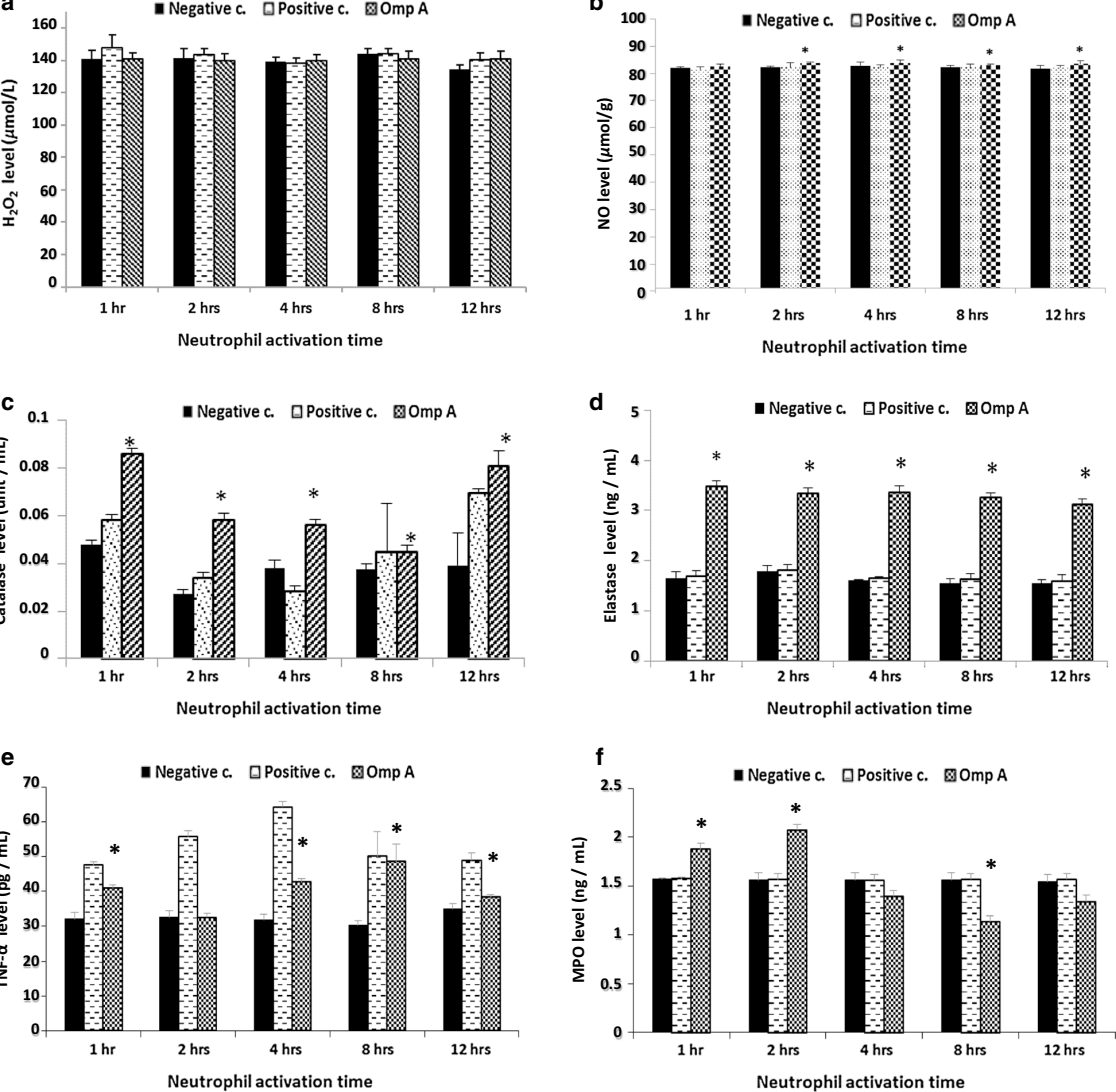
The OmpA-like protein BCAL2958 interferes with neutrophils activity. **a** Level of hydrogen peroxide produced by neutrophils in response to OmpA at different incubation times; **b** Level of nitric oxide produced by neutrophils in response to OmpA at different activation times; **c** Level of catalase produced by neutrophils in response to OmpA at different activation times; **d** Level of elastase produced by neutrophils in response to OmpA at different activation times; **e** Level of TNF-α produced by neutrophils in response to OmpA at different activation times; **f** Level of MPO produced by neutrophils in response to OmpA at different activation times. *Asterisks* significant as compared to negative control at P < 0.05

After incubation with BCAL2958 protein, neutrophil secreted TNFα, elastase and NO secretion increased significantly in all incubation times, suggesting an activation of the neutrophils (Fig. 5b, d, e).

BCAL2958 enhanced also MPO secretion from neutrophils, mainly during the first 2 h of incubation (Fig. 5f). In contrast, no significant change in H_2_O_2_ concentration was observed (Fig. 5a). Neutrophils azurophilic granules contain a rich supply of the enzyme MPO that in combination with H_2_O_2_ and chloride constitutes a potent anti-microbial system (Klebanoff 2005). Therefore, our results suggest that due to high levels of secretion of MPO during the first 2 h of incubation, the neutrophils released H_2_O_2_ could have been consumed directly into other forms of oxygen radicals.

Significant increases of catalase were also observed after all neutrophil activation times in response to BCAL2958 (Fig. 5c). The catalase detoxifies H_2_O_2_ to oxygen and water, reducing also the H_2_O_2_ levels produced by the neutrophils (Roos et al. 1980).

## Discussion

Despite the emergence of novel therapies (Chmiel et al. 2014), bacterial lung infections remain the main cause of morbidity and mortality in CF patients, with *P. aeruginosa* as the leading pathogen. For this pathogen, eradication is hardly achieved in chronically infected patients by antibiotic treatments (Ciofu et al. 2013; Chmiel et al. 2014), while early infection eradication is more easily achieved (Schelstraete et al. 2013). Therefore, novel approaches preventing either the adherence or the colonization of the CF lung by bacterial pathogens are envisaged as the most promising eradication strategies (Regan and Bhatt 2014). In the case of Bcc infections, no objective guidelines for eradication strategies are available, as these pathogens are inherently resistant to the majority of antibiotics (Regan and Bhatt 2014).

Predominant immunogenic components of pathogenic bacteria are potential candidates for the design of novel serological diagnosis tests and the development of strategies for efficient immune protection and eradication. OmpA-like proteins, which are important immunogenic components of the outer membrane of gram-negative bacteria, are promising candidates for such purposes. This is the case of the OmpA-like proteins such as the 17 kDa OmpA from *B. cenocepacia*, OmpA from *E. coli*, OmpA from *Shigella flexneri* 2a and the OprF from *P. aeruginosa* (Makidon et al. 2010; Guan et al. 2015; Pore and Chakrabarti 2013; Baumann et al. 2004). The knowledge of the potential immunogenicity of these OmpA-like proteins prompted us to investigate the *B. cenocepacia* J2315 BCAL2958 protein, previously identified by our group when analysing the relative virulence of a *B. cenocepacia* random plasposon mutant library (unpublished results).

Bioinformatics analysis of the BCAL2958 deduced amino acid sequence revealed the presence at the protein C-terminus of the conserved peptidoglycan-binding (critical for the noncovalent interaction with the underlying PG layer in the periplasm (De Mot and Vanderleyden 1994; Koebnik et al. 2000), while its N-terminal part exhibited a low or null similarity to the other outer membrane proteins analysed. OmpA-like proteins typically have highly variable and heterogeneous N-terminal regions.

Inspection of the genomes of members of the Bcc with completed and publicly available genome sequences revealed several putative ORFs encoding OmpA-like proteins. In particular, all the inspected Bcc genomes revealed the presence of BCAL2958 orthologues with an identity higher than 96 %. In a previous work using an immunoproteomic approach with a pool of serum samples from Bcc-infected CF patients, Shinoy et al. (2013) observed that OmpA-like proteins were immunoreactive and were present in strains of *B. cenocepacia* and *B. multivorans*. In the present work, we furthered this observation, showing that the BCAL2958 protein is expressed by 12 strains belonging to 7 different *Burkholderia* species isolated from CF patients with different geographical origins, based on western blot experiments using an antibody raised against *B. cenocepacia* J2315 BCAL2958. Conservation of this surface antigen among Bcc strains is critical for its possible use either as a diagnostic reagent or a vaccine. OmpA-type porins from several other bacterial species have been shown to be highly immunogenic, inducing specific humoral and cytotoxic responses, even in the absence of adjuvants (Jeannin et al. 2005). In this work, we found that the BCAL2958 protein of *B. cenocepacia* J2315 is recognized by all the four CF serum samples tested, revealing that this protein can elicit a humoral immune response in CF patients.

Recombinant purified forms of OmpA has been shown to activate both macrophages (Soulas et al. 2000), dendritic cells (DCs) (Jeannin et al. 2000; Lee et al. 2010) and neutrophils (Mantovani et al. 2011) in a receptor-dependent manner, suggesting that OmpA functions as a molecular pattern that activates the immune system. In CF lung disease, neutrophils are the most rapid and predominant innate immune cell type to transmigrate into the infected CF airway compartment (Hector et al. 2016). Previously, we observed colocalization of Bcc and neutrophils by immunofluorescence staining of Bcc infected CF lung tissue samples who underwent transplantation, suggesting Bcc persistence within neutrophils in CF patients (Sousa et al. 2007). Therefore, we analysed the effect of BCAL2958 on human neutrophils. Neutrophils have surface receptors that have evolved to recognize and bind to surface bacterial constituents and trigger the cell to engulf the bacterium and induce the secretion of biologically active molecules, such as cytokines (e.g. TNFα) and MPO (Kumar and Sharma 2010). Our results indicate that BCAL2958 protein induces the secretion of neutrophil TNFα, elastase, catalase and NO, suggesting an activation of the human neutrophils. The *K. pneumoniae* OmpA was also shown to up-regulate the secretion of cytokines (e.g. TNF-α, IL-1β, IL-10, and IL-12) from murine and human macrophages (Jeannin et al. 2005). OmpA from *E. coli* was shown to be the direct target of neutrophil elastase (Belaaouaj et al. 2000).

Stimulation of neutrophils with outer membrane vesicles (OMV) from serogroup B *Neisseria meningitidis* was reported to led to the production of TNF-α, IL-8, MIP-1α and MIP1-β, and to the activation and recruitment of monocytes/macrophages at the site of inflammation. Thus, neutrophils may influence the phenomenon of macrophage differentiation into pro-inflammatory or anti-inflammatory subtype (Lapinet et al. 2000). TNF-α released by activated neutrophils also plays an important role in the maturation of DCs (Bennouna and Denkers 2005). Neutrophils are also reported to enhance specific, adaptive T and B cell responses by enabling the differentiation of monocytes and DCs into professional antigen-presenting cells (Cerutti et al. 2013; Mantovani et al. 2011). Therefore, the observed effect of BCAL2958 on neutrophil activation and on the most probable activation of other immune host cells, such as macrophages and T cells will be interesting to further enlighten the type of immune response of CF patients to the protein BCAL2958.

In conclusion, the presence of genes encoding BCAL2958-like proteins within Bcc genomes, together with the confirmed expression of the protein by multiple Bcc members and its immunoreactivity with serum samples from CF patients infected with Bcc renders this protein as a potential candidate for the development of novel strategies for immunoprotection against Bcc infections and/or a novel diagnostic method for early detection of Bcc infections. Future experiments involving studies on the immunization of mice with purified BCAL2958 will give critical information on this subject.
